# Phthalocyanine and Its Formulations: A Promising Photosensitizer for Cervical Cancer Phototherapy

**DOI:** 10.3390/pharmaceutics13122057

**Published:** 2021-12-02

**Authors:** Lucimara R. Carobeli, Lyvia E. de F. Meirelles, Gabrielle M. Z. F. Damke, Edilson Damke, Maria V. F. de Souza, Natália L. Mari, Kayane H. Mashiba, Cristiane S. Shinobu-Mesquita, Raquel P. Souza, Vânia R. S. da Silva, Renato S. Gonçalves, Wilker Caetano, Márcia E. L. Consolaro

**Affiliations:** 1 Department of Clinical Analysis and Biomedicine, Universidade Estadual de Maringá, Maringá 87020-900, PR, Brazil; lucimaracarobeli@gmail.com (L.R.C.); lyvia.fmeirelles@gmail.com (L.E.d.F.M.); gabizago@gmail.com (G.M.Z.F.D.); edilsondamke@gmail.com (E.D.); mariavitoriafesouza@gmail.com (M.V.F.d.S.); nlmari95@gmail.com (N.L.M.); kayanehmashiba@gmail.com (K.H.M.); cristianeshinobu@gmail.com (C.S.S.-M.); raquelpantarotto@gmail.com (R.P.S.); vaniasela@gmail.com (V.R.S.d.S.); 2 Department of Chemistry, Universidade Estadual de Maringá, Maringá 87020-900, PR, Brazil; rsonchini@gmail.com (R.S.G.); wcaetano@uem.br (W.C.)

**Keywords:** phthalocyanine, uterine cervical neoplasms, photochemotherapy, in vitro, in vivo

## Abstract

Cervical cancer is one of the most common causes of cancer-related deaths in women worldwide. Despite advances in current therapies, women with advanced or recurrent disease present poor prognosis. Photodynamic therapy (PDT) has emerged as an effective therapeutic alternative to treat oncological diseases such as cervical cancer. Phthalocyanines (Pcs) are considered good photosensitizers (PS) for PDT, although most of them present high levels of aggregation and are lipophilic. Despite many investigations and encouraging results, Pcs have not been approved as PS for PDT of invasive cervical cancer yet. This review presents an overview on the pathophysiology of cervical cancer and summarizes the most recent developments on the physicochemical properties of Pcs and biological results obtained both in vitro in tumor-bearing mice and in clinical tests reported in the last five years. Current evidence indicates that Pcs have potential as pharmaceutical agents for anti-cervical cancer therapy. The authors firmly believe that Pc-based formulations could emerge as a privileged scaffold for the establishment of lead compounds for PDT against different types of cervical cancer.

## 1. Introduction

Cervical cancer is one of the most common types of cancer in women worldwide with an estimated 604,000 cases and 342,000 deaths worldwide in 2020 [1,2]. Globally, it represents the fourth most frequent type of cancer and the fourth leading cause of cancer death in women despite being considered nearly completely preventable because of the highly effective primary (human *Papillomavirus*-HPV vaccine) and secondary (screening) prevention measures available [1,2,3,4]. Specifically, since 2006 HPV vaccination has been available for the prevention of cervical cancer. However, even if high coverage vaccination can be quickly implemented, a substantial effect on disease burden will be seen after three to four decades and the short-term impact will require cervical screenings for older cohorts not covered by the HPV vaccine [3,4]. These preventable measures have not been equitably implemented across and within countries [2,5]. This is evidenced by the data that around 90% of cervical cancer cases occur in low- and medium-income countries (LMICs), where the incidence and disease-specific mortality continue to increase [2,6]. Indeed, the data show that rates remain disproportionately high in developing versus developed countries (18.8 vs. 11.3 per 100,000 for incidence; 12.4 vs. 5.2 per 100,000 for mortality) [2]. Additionally, analyzing data from different countries, cervical cancer is the most common type of cancer in 23 countries and the leading cause of cancer death in 36 countries, especially in sub-Saharan Africa, Melanesia, South America, and South-Eastern Asia [2]. All these data highlight that cervical cancer is a striking example of a global health disparity [6].

Surgery and radiation therapy are the most common options for the treatment of invasive cervical cancer. The type of treatment will depend on factors such as the stage of the disease; the size of the tumor; and personal factors, such as age and desire to have children [7]. The clinical staging of cervical cancer follows the guidelines of the International Federation of Gynecology and Obstetrics (FIGO) [8], and for treatment, patients can be grouped in early-stage disease; locally advanced disease; and advanced disease, which includes persistent, recurrent, or metastatic disease [9]. For early-stage disease, surgery is the primary treatment modality with high cure rates and overall 5-year survival up to 92% [10]. Surgery or radiation associated with chemotherapy can be used [11,12]. In advanced cervical cancer, the main therapeutic option is chemoradiation with a platinum-based agent [13], and chemotherapy before surgery or chemoradiation that is associated with inferior outcomes compared to concurrent chemoradiation [14]. Specifically, chemotherapy can work as an adjuvant when used immediately after the primary treatment of the tumor by surgery or radiation therapy. It can also be a neoadjuvant when performed before local treatment with the aim of reducing the tumor size and providing adequate conditions for the subsequent surgical and/or radiotherapy treatment. In addition, there is concomitant chemo-radiotherapy that is administered simultaneously with radiotherapy to enhance the treatment effect and increase the patient’s survival; however, the side effects tend to be worse [11]. The most commonly used chemotherapeutics for the treatment of cervical cancer include cisplatin, carboplatin, paclitaxel, topotecan, and gemcitabine. Cisplatin is the most effective cytotoxic agent against metastatic cervical cancer; in addition, it increases sensitivity to radiotherapy [15]. The side effects of chemotherapy depend on the type of drug, the dose administered, and the duration of treatment. These effects are temporary and can include nausea and vomiting, loss of appetite, mouth sores, hair loss, inflammation in the mouth, infection, bleeding or bruising after minor cuts or injuries, and shortness of breath [11].

The use of concomitant chemoradiotherapy improves the 5-year survival rates of patients with advanced cervical cancer. Nevertheless, once post-treatment failure occurs, the 1-year survival rates are less than 20%. Therefore, the occurrence of local recurrence and distant metastasis is still very common in these patients, significantly decreasing survival rates [16]. Therefore, early-stage and locally advanced disease can be cured, but women with advanced or recurrent disease present a poor prognosis. Despite these alarming facts, efficient treatment methods are still lacking. This underscores the need for different treatment strategies, particularly for advanced cervical cancer, which is still the leading cause of death in developing nations with limited access to care [13]. 

In the last 20 years, little progress has been made on the development of novel therapeutic modalities for preinvasive (cervical intraepithelial neoplasia-CIN) or invasive cervical cancer [17]. Since the first use of a hematoporphyrin derivative in conjunction with red light irradiation to kill tumor cells in 1975, photodynamic therapy (PDT) has attracted attention as a promising strategy for cancer treatment [18]. The main elements of PDT are a photoactive drug (PS), light at an appropriate wavelength, and molecular oxygen [9,10]. Light excites the PS to its triplet state, interacting with the oxygen present in the tissue to produce reactive oxygen species (ROS) via a type I or type II mechanism. This interaction results in the production of free radicals [11] or singlet oxygen (^1^O_2_) [12], respectively. PDT is an attractive treatment modality for cancer because the PS, light, and oxygen separately do not present any toxic effects on the body, unlike chemotherapy, which induces systemic toxicity and radiotherapy that damage neighboring normal tissues [19]. PDT is currently applied against numerous cancers, and it is clinically approved in the United States for esophageal and non-small cell lung cancers. Additionally, PDT is being tested in clinical trials for several other types of cancers, including glioma, pediatric brain cancer, prostate cancer, head and neck cancer, cervical cancer, pancreatic cancer, breast cancer, and oral cancer [18]. For cervical cancer, the Photocure system (Cevira^®^), a multinational Phase III trial, was developed to treat persistent oncogenic HPV and cervical precancerous lesions. This technology is based on the hexylaminolevulinate (prodrug of protoporphyrin IX) that is administered locally to the cervix by a drug and light delivery device. Cevira has been shown in patients to be efficient and safe [20]. Overall, PDT has the following advantages in the treatment of CIN and invasive cervical cancer: localized treatment through light application, HPV inactivation if properly applied, selectivity of action in dysplastic and neoplastic cells, the possibility of repeated use without leading to cumulative toxicity, the possibility of combination with chemotherapy and radiotherapy to obtain better results, and the improvement of antitumor immunity helping in tumor elimination as well as tumor control in the medium and long term [21]. Moreover, PDT does not induce systemic side effects and can be repeated several times without tumor resistance, with a short delay between consecutive therapies [22]. Finally, PDT does not require anesthesia in many patients and generally does not cause bleeding, which makes its use applicable to the outpatient setting [17].

A PS should ideally have the following key features: (1) be a pure compound; (2) present a long life of the triplet reactive excited species generated by the photoactive pathways (greater than 500 ns), enough to react with molecular oxygen and induce ROS production; (3) present a strong absorption peak in the red to near-infrared spectral region (NIR) (between 650 and 800 nm) because the absorption of single photons with wavelengths longer than 800 nm does not provide enough energy to excite oxygen to its singlet state. Additionally, the NIR is used because most tissues transmit light reasonably well in this spectral domain; (4) present a substantial triplet quantum yield leading to good production of ROS upon irradiation; (5) no dark toxicity and relatively rapid clearance from normal tissues, thereby minimizing the side effects of PDT; and (6) present good solubility and biocompatibility [23,24,25,26,27,28,29]. Since the discovery of PDT, continuous efforts have been made to identify new ideal PS with this set of characteristics.

Historically, the first approaches to PDT are found in ancient texts such as the Egyptian medical treatise Ebers Papyrus and the Atharvaveda from the Hinduism. Despite this background, the observation of photochemical sensitization of tissues was first performed in detail by Raab (1900) in Germany and shortly afterwards by Von Tappeiner (1900), who coined the term “photodynamic action” to describe the treatment of skin tumors by using topical administration of eosin combined with sunlight [30]. The majority of PS currently in use for PDT are cyclic tetrapyrrolic structures: porphyrins and their analogs; chlorins; bacteriochlorins; and phthalocyanines (Pcs). Among them, Pcs include a variety of substances considered good PS for cancer PDT, with activation in the range from 670 to 690 nm and well-defined tissue penetration in the target cells. Since Pcs absorb long-wavelength light strongly; localization is in their cells; and they have triplet yield, among other features, they can be used in small doses [31]. Despite their interesting optical properties in the NIR, the applications of these hydrophobic planar molecules were not fully performed because of poor solubility, which is crucial in order to reach the therapeutic target and also a tendency to form aggregates. Aggregation causes the formation of exiplexes upon light absorption, quenches excited state lifetimes, and diminishes photophysical properties. More specifically, the formation of aggregates in solution decreases the capacity to produce singlet oxygen because the photochemical activity is exclusively related to monomer species. Thus, aggregates decrease not only the photoactivity of the Pc but also limit the access to the neoplastic cells, affecting its bioavailability [32,33,34]. Additionally, depending on the dose, some Pcs such as ZnPc can cause skin phototoxicity in patients [32]. As result, several studies sought for structural variations in Pcs molecules, such as incorporation of appropriate aromatic substitutions or an element in the molecule’s core structure to reduce the degree of hydrophobicity. These attempts led to the development of easy synthetic routes for a new generation of Pcs with improved solubility and reduced aggregation [34,35]. Another option to increase the solubility and bioavailability of hydrophobic Pcs and their derivatives is a formulation using various drug delivery systems (DDSs) such as nanoemulsions and liposomal, among others [36], which present themselves as feasible approaches to increase Pcs’ biocompatibility. Despite these advances and encouraging results, Pcs are not yet approved as PS for invasive cervical cancer PDT.

This review presents an overview of the pathophysiology of cervical cancer that may influence the interpretation of results from studies with different formulations using Pcs for PDT against cancer. We also review the photophysical properties of Pcs, the in vitro effects and antitumor mechanism of action in cell cultures, and the in vivo effectiveness as anticancer agents in a tumor-bearing mice model and clinical trials reported in the last five years. There is also an emphasis on various strategies to enhance the functions of Pcs, showing the great promise of this class of functional dyes as advanced PS for PDT of CIN and cervical cancer.

## 2. Cervical Cancer

### 2.1. Basic Concepts

The endocervical canal is lined with the stratified squamous epithelium and columnar epithelium, which cover the ectocervix and endocervix, respectively. The transition zone between these cells is called the squamocolumnar junction. Any premalignant cellular transformation occurs mostly at the squamocolumnar junction and is closely associated with high-risk HPV types. The premalignant changes or dysplasia of squamous cells in the cervical epithelium are collectively known as CIN [6,8]. CIN grade 3 (CIN 3) precedes the development of invasive cervical cancer by 10–20 years, making cervical cancer preventable if these lesions are detected and effectively treated [37]. Most of the therapeutic methods for the treatment of CIN3 can be divided into two basic groups: (a) destructive methods (diathermocoagulation, cryodestruction, and laser evaporation) and (b) removal of the pathologic tissue (surgical, laser, or electrosurgical excision). Different treatment modalities, including cold-knife excision, electrocautery, cryosurgery, laser ablation, and large-loop excision of the transformation zone (LLETZ), have shown high success rates [12]. However, the most common adverse effects of such treatment are hemorrhage, traumatization of subjacent tissues with a formation of rough scraps, stenosis, and stricture of the cervical canal. Changes in the anatomic structure of the cervix uteri lead to the loss of functionality; specifically, a reduction of cervical secretions results in a decrease in the probability of conception, an increase in spontaneous abortion, an increase in the level of perinatal mortality, and impairment to normal delivery [38]. Indeed, the optimal management of CIN3 requires development and introduction of novel therapeutic approaches. On the other hand, mild and moderate lesions (CIN1 and CIN2), which may precede CIN3, are generally not treated but require periodic long-term screening to monitor its development or disappearance [39]. However, considering that the accuracy of Papanicolaou (Pap) smear in diagnosing cervical precancerous lesions is low, sometimes contradictory results between biopsy and Pap smear are reported, which hinders the clinical decision concerning whether or not to adopt treatment as well as the type of treatment.

The main types of invasive cervical cancer are squamous cell carcinoma (SCC) (around 70% of cases), adenocarcinoma (AC) (around 25% of cases), adenosquamous cell carcinoma (ASC), neuroendocrine-endocrine cancer, glassy cell carcinoma, and undifferentiated cancer [40]. It is common knowledge that the most important cause of cervical cancer is persistent HPV infection. HPV is detected in 99% of cervical tumors [41], with HPV-16 being the most prevalent type, followed by HPV-18; together, these types account for around 70% of SCC and over 80% of AC cases [42,43]. However, oncogenic or high-risk HPV types (hrHPV) are virtually necessary but not sufficient causes for cervical cancer development [41]. Most hrHPV infections are eventually cleared, but some cases progress to high-grade squamous intraepithelial lesions (HSIL) (10% of HPV infections) and invasive cervical cancer (<1% of HPV infections) [44,45,46,47]. The reasons for this variable natural history are poorly understood [48], but it is generally assumed that other causes or cofactors are important for the development of neoplasia in HPV-infected women [44,46,49]. Other important cofactors to cervical cancer development include immunosuppression (particularly human immunodeficiency virus), smoking, parenthood (a higher number of full-term pregnancies increases risk), use of oral contraceptive [50], and sexually transmitted infections (such as chronic infection by *Chlamydia trachomatis* [48]).

HPVs are small, non-enveloped, double-stranded DNA viruses that exhibit a strict tropism for cutaneous and mucosal (e.g., oropharynx, anogenital tract) epithelium [51,52]. At present, more than 200 different types of HPV have been identified [53] and approximately 54 are able to infect the reproductive tract mucosa [54], with 12 oncogenic types classified as group 1 carcinogens [55]. The most common hrHPV types include HPV-16, 18, 31, 33, 35, 39, and 45 [54]. HPV infects the actively proliferating, undifferentiated basal keratinocytes of the stratified squamous cervical epithelium that are thought to become exposed through a microlesion [56]. Two viral promoters, early (E) and late (L), regulate viral gene expression and are active at different stages in the life cycle [57]. The establishment and maintenance of HPV genomes in infected cells are associated with the expression of early genes E5, E6, and E7, which encode their respective oncoproteins, as well as the replication proteins E1 and E2. In the basal undifferentiated cells, viral genomes are subsequently maintained at low copy number by replicating along with cellular DNA [52,58]. During cell division, basal cells leave the basal layer, migrate to the suprabasal region and begin to differentiate. At this stage, the viral genome is divided among the daughter cells, which, when migrating to the upper layers of the epithelium during the differentiation process, continue with the active cell cycle. In differentiated layers of the epithelium, the viral DNA is packaged into new capsids and virus progeny is released from the upper layer of the epithelium [57,59,60,61,62,63]. 

The development of cervical cancer is associated with loss of regulation of the HPV production cycle, an event observed in persistent hrHPV infections, which tend to integrate their genome to the host cell [46,49]. During the integration process, the viral genome may lose the E4 gene and part of the E2 gene, which play roles in controlling the transcription of other viral genes. The consequence of the loss of E2 function is an increase in the expression of the E6 and E7 genes. In this scenario, there is no maturation of host cells and no production of new viral particles [46,64,65]. Thus, HPV oncogenic potential depends on the expression of E6/E7 genes whose products, the E6 and E7 proteins, have as their main target the p53 and pRb proteins of the host genome, inducing lack of control of the cell cycle [66,67,68,69,70,71,72].

In face of the complexity of cervical carcinogenesis and the different types of hrHPV involved, it is important to correctly apply in vitro and in vivo models that represent this complexity for testing molecules or biologics for anti cervical cancer drug development.

### 2.2. Human Cervical Cancer-Derived Cell Lines to In Vitro and In Vivo (Tumor-Bearing Mice) Studies

Human tumor-derived cell lines present a historically important role in the discovery and development of new therapeutic agents against cancer. In vitro studies using immortalized human cancer cell lines provide controlled conditions to assess the effectiveness of drugs and provide unrestricted availability of human-derived material [73]. For the discovery of new anti cervical cancer drugs, human tumor-derived cell lines are even more important, since no animal model of HPV infection exists [74]. This is due to the fact that Papillomaviruses are strictly species-specific and even in experimental conditions do not infect any other host than their natural one. There is only one known case of cross infection between horses and other equidae by bovine papillomavirus (BPV) types 1 and 2. Additionally, HPV is strictly tissue-restricted and cannot be propagated in vitro. Consequently, to evaluate tumors triggered by HPV on in vivo animal studies, an experimental model of tumor induction could be performed where researchers would inoculate the human cervical cancer-derived cell lines through subcutaneous injection in mice, for example, tumor-bearing mice. Therefore, major knowledge regarding the HPV life cycle, the oncogenicity, and the synergy with cofactors was first established in vitro or by animal papillomaviruses in vivo. However, DNA sequences of HPV-16, -18, -45, and -68 have been found in cell lines established from human cervical carcinomas [75,76,77]. Since animal models for cellular transformation by HPV are not available, cervical cancer cell lines are unique tools for understanding the biology and tumor response as well for testing small molecules or biologics for anti-cancer drug development.

At present, immortalized cervical cancer cell lines available in major cell repositories, including American Type Culture Collection (ATCC) and European Collection of Authenticated Cell Cultures (ECACC), represent six cervical cancers (not considering HeLa-derived cell lines) [78], reflecting the two main types of cervical cancer (SCC and AD) and the two main types of hrHPV involved in cervical carcinogenesis (HPV-16 and 18) as well as HPV-45 and HPV-68. Still, the availability of a series of HPV-positive (Hela, SiHa, Caski, MS751, ME-180, and C-4I) and HPV-negative (C-33A) human cervical carcinoma cell lines provides the opportunity to evaluate whether a new drug candidate is effective or not in both scenarios. Table 1 summarizes the main human immortalized cervical cancer cell lines and their characteristics.

## 3. Photodynamic Therapy (PDT)

### 3.1. Principles and Applications

**Scheme 1:**
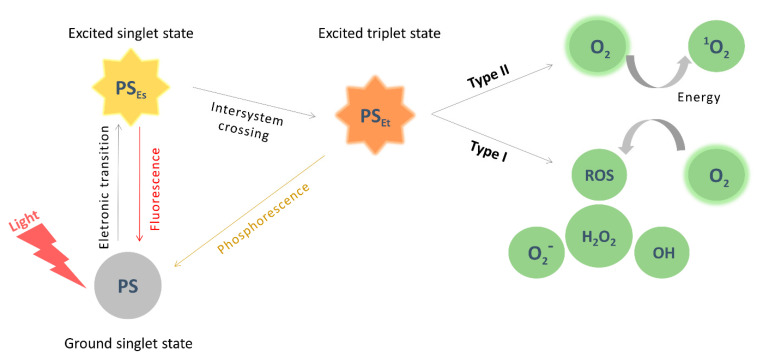
Photodynamic reaction mechanism.

PDT is a promising treatment modality. The light is absorbed by the PS either systemically, locally, or topically for the treatment of a variety of pathologies such as oncological [79], cardiovascular [80], dermatological [81], ophthalmic [82], bacterial, viral, and parasitic infections [83]. Possibly the main use of PDT in the world up until now has been its use in ophthalmology, i.e., in the treatment of wet age-related macular degeneration and PVC [82]. Photoactivation of the PS in the presence of oxygen and a target cell or tissue are necessary for PDT. Specifically, the PS is excited by light inducing two photochemical mechanisms (type I and type II). In Type I, the PS in its excited triplet state interacts with a biomolecule transferring or acquiring one electron/hydrogen via the radical mechanism, leading to the generation of ROS, including superoxide anions (O_2_^−^^●^), singlet oxygen (^1^O_2_), hydroxyl radicals (HO^●^), and hydrogen peroxide (H_2_O_2_), that attack cellular targets and damage cellular components. However, original free radicals can cause cellular damage directly [19,36,83,84]. In Type II, the direct energy transfer occurs from the PS in the triplet excited state to the oxygen in the fundamental triplet state, producing cytotoxic singlet oxygen [19,83,84]. Singlet oxygen presents a short lifetime in a time-scale of microseconds, but a sufficient concentration of highly cytotoxic singlet oxygen can induce irreversible cell damage [36]. Furthermore, the location of the PS changes over time, and therefore, soon after the application, it is located in the vasculature and PDT can mainly result in the closure of blood vessels. Although both types of reactions (Type I and II) take place at the same time, most PS used for anti-cancer PDT are believed to operate via the Type II rather than the Type I mechanism [84,85,86,87,88], possibly due to ROS generation via Type II chemistry being mechanistically much simpler than via Type I. After photochemical reaction, light-mediated destruction of the PS can occur [89] or the PS can return to its ground state without modifications and can be prepared to repeat the excitation-energy transfer process multiple times [23,90,91,92] (Scheme 1).

As mentioned earlier, in order to ensure high effectiveness in PDT, is important that the PS present some outstanding characteristics, such as low (or no) toxicity to normal tissue, high therapeutic selectivity, non-prolonged photosensitivity, long circulation time in plasma, and enhanced accumulation in target tissue [93].

Several types of fiber tip or light applicator, e.g., lens, cylindrical diffuser, and spherical diffuser, have been developed to facilitate the light delivery and to optimize the light transmission and distribution in the lesion site. Lens-based fiber-optic light distributors are very useful for the frontal irradiation of superficial lesions as the optical lens can offer better light uniformity and beam expansion. The thin flexible fiber with microlens tip is particularly suitable for endoscopic applications. Recently, macrobendings (two perpendicular mode scramblers) have been used in PDT fibers to improve the uniformity of light beam [94]. The aforementioned Cevira technology clearly emphasizes the importance of developing light distribution devices specifically dedicated to the cervix [20].

### 3.2. PDT on Cancer

**Figure 1:**
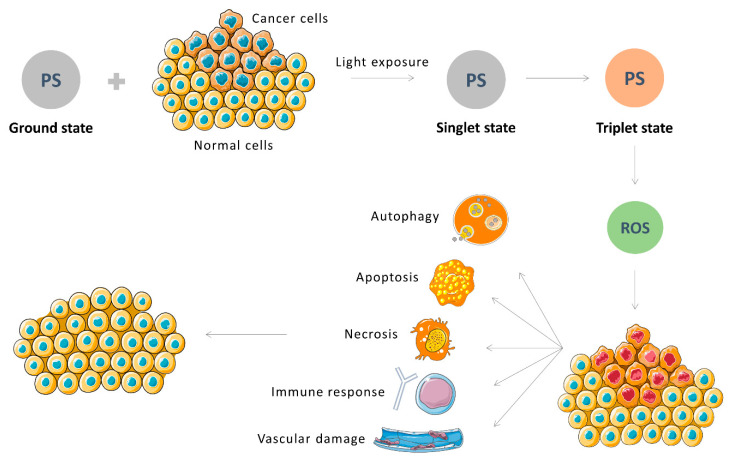
Photodynamic therapy (PDT) for cancer treatment.

PDT effectiveness on cancer treatment is closely related to the high selectivity of PS accumulation rate in the cancer tissue. The high selectivity in PDT comes from a partially selective accumulation of the drug combined with the selective application of light. Consequently, the short lifetime of ROS especies can be enough to generate the photodynamic activity and oxidative damage to endogenous biomolecules (e.g., proteins and DNAs), leading to cell death via apoptosis, necrosis, or autophagy-associated mechanisms [95,96,97,98,99]. ROS can also be indirectly related to stimulation of the transcription and release of inflammation mediators [100]. In addition, an elevated ROS level leads to cellular oxidation process by damaging plasma membranes and cell organelles and subsequent alteration in permeability and transport function between intra and extracellular media [101]. PDT targets include tumor cells, tissue microvasculature, and the host’s inflammatory and immune systems. The combination of all these components is necessary for the long-term control of the tumor [36,102,103] (Figure 1).

The extent of damage and the cell death pathways involved depend on the type and concentration of the PS, its subcellular location, the energy applied, and the type of tumor [104]. The uptake of the PS by cancer cells as well as the localization within or on the cell surface depend on its chemical characteristics [105,106,107]. Hydrophobic PS tend to diffuse rapidly into plasma membranes and gather in intracellular membrane structures such as mitochondria and endoplasmic reticulum (ER). Hydrophilic PS tend to be internalized by endocytosis or transport by lipids and serum proteins [79,84]. After internalization, the intracellular location of the PS can be highly specific or quite broad [79,84] and has been reported to include the ER, mitochondria, Golgi complex, lysosomes, and plasma membrane [79,84]. Furthermore, it is very important that the PS does not accumulate in cell nuclei in order to limit DNA damage and prevent the appearance of genetically resistant cells [79]. Importantly, the location of the PS in cells is critical to determine the cell death pathway resulting from PDT and consequently the cell response to photodamage [105,106,107]. In this sense, it is very important to understand the preferential subcellular location of the PS in order to predict its cytotoxic potential [105,106,107,108]. The best described cytotoxic effects of organelle-specific PDT are in mitochondria, lysosomes, and ER [104].

As discussed above, PDT induces cell death via necrosis, apoptosis, or autophagy. The predominant mechanism depends on the PS and light dose, cell type, and subcellular localization [109,110]. The production of ROS at the mitochondrial, lysosomal, or ER location of the PS can directly initiate apoptotic cell death [111], which can be defined as a type of programmed cell death. Apoptosis is an induced and regulated process that activates a family of proteins (known as caspase) as well as precise cellular events, which degrade nucleic and polypeptide materials [103]. Necrosis is a non-programmed cell death and is more often observed when the PS site of action is in the plasma membrane and/or when it is activated with high-energy doses [79]. In order to suppress undesired damage to normal tissues, this effect should in general be avoided [112]. On the other hand, necrosis is an unscheduled cell death and is most often observed when the PS localizes in the plasma membrane and/or when it is activated by high doses of energy [79]. In the latter case, it is called accidental necrosis, and in order to prevent damage to normal cells, it must be avoided [112] with the use of appropriate doses of energy. Some reports have suggested that a pronounced inflammatory response resulting from necrotic cell death after PDT is important because it aids the immune-stimulating function of PDT [113]. Finally, the autophagic pathway is an evolutionarily old cytoprotective mechanism that aims to reestablish homeostasis [105,114,115]. This is a death pathway that appears not to be limited to the subcellular compartment in which PS accumulates [105,114]. The induction of autophagy appears to be a common response in PDT protocols [114], despite the role of autophagy as a factor in PDT-induced cell death is not yet clear. Despite this, PDT-induced autophagy appears to play a prosurvival role in apoptosis-competent cells and possibly a prodeath role in apoptosis-defective cells [105,114]. Additionally, when impaired or insufficient autophagy is triggered, induction of cell death is the most common result observed [105,114,115].

Although PDT has been proven as an effective broad-spectrum therapeutic, and it has been used with relative success in the field of oncology specially for the treatment of superficial oncologic lesions [116,117]. Recent advances in fiber optic technology have enabled the use of PDT far beyond its dermatological applications. Light sources such as lasers can be coupled with fiber optic systems to allow deep or difficult-to-treat tumors to be treated with PDT, such as urinary bladder, digestive tract, and brain and breast tumors [118]. Moreover, PDT can be used in association with other therapeutic techniques such as surgery or chemotherapy. In the case of surgery for tumor resection, PDT can help to destroy any remaining cancer cells after surgery. Several trials have demonstrated synergistic effects by combining PDT with low-dose chemotherapeutics [118,119,120]. For example, the PS can overcome the multi-drug resistance of the chemotherapeutic, whereas the chemotherapeutic can address the limitations of light penetration and hypoxia-related resistance in PDT [120].

Regarding the light power, it was previously established that the fluence rate affects the PDT tumor response. A high fluence rate decreases or even totally inhibits tumor control. The influence of fluence rate is not restricted to cytocidal effects, but it can also be seen in sublethal conditions such as vascular permeability. This has been shown to be true in pre-clinical and clinical settings. Therefore, this is an extremely important parameter in PDT in vitro and in vivo studies [121].

Several PS are in clinical use or undergoing clinical trials to treat cancer. Among them, the hematoporphyrin derivative (Photofrin), a first-generation PS with prolonged cutaneous phototoxicity can be highlighted. Photofrin is a HPLC-purified form of hematoporphyrin and has already been approved by the Food and Drug Administration (FDA) for the palliative treatment of obstructive diseases such as lung and esophageal cancers. Other PS can still be mentioned. Among them, a second generation have improved pharmacokinetics and reduced skin photosensitivity, such as benzoporphyrin derivative (BPD), 5-ethylamino-9-diethyl-aminobenzo [a] phenothiazine chloride (EtNBS), silicon phthalocyanine (Pc4), m-tetrahydroxyphenylchlorine (mTHPC), mesochlorin e6 (Mce6), and mono-l-aspartylchlorin e6 (NPe6). Additionally, there are several other PS at different stages of development [122].

### 3.3. Pcs as Photosensitizers for Cancer PDT

Pcs are referred to as tetrabenzotetraazaporphyrins and belong to the group of 2nd generation PS. Pcs are synthetic macromolecules related to tetra-aza porphyrins (porphyrazines) and have a benzene ring fused to each of four pyrrole subunits, which are linked by four nitrogen atoms (like porphyrazines macrocycle) instead of four bridging carbon atoms in porphyrin macrocycle [123]. Pcs are usually prepared in the form of complexes with metal cations such as zinc, aluminum, and gallium, co-ordinated to the centre of the macrocycle. This is due to the fact that during synthesis transition, metal cation helps to close the ring to easily form a macrocycle. The photophysical properties of Pcs are strongly influenced by the presence and nature of the central metal ion and result in metallophthalocyanines (MPcs) with high singlet oxygen quantum yields [31,124,125,126,127,128,129].

**Scheme 2:**
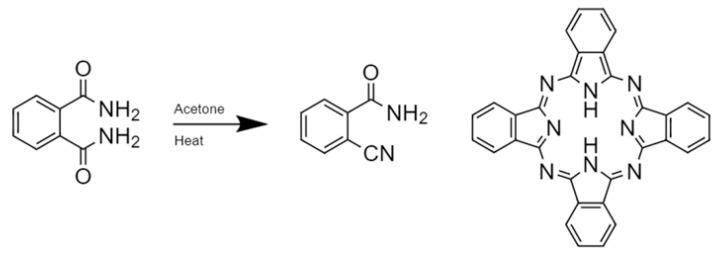
Synthesis of the first metal-free phthalocyanine as a product during the preparation of ortho-cyanobenzamide from phthalamide in acetone.

Considering that Pcs are flexible and stable compounds that improved light absorption capabilities, they have been used but not limited to chemical sensors and semiconductors, in nonlinear optics and as PS [130,131]. The first metal-free Pcs were discovered by Braun and Tcherniac in 1907 as a product during the preparation of ortho-cyanobenzamide from phthalamide in acetone (Scheme 2) [103,132,133]. Pcs can be obtained by the classic template reactions starting from diverse precursors, such as phthalonitrile (PN), o-cyanobenzamide, 1,3-diiminoisoindoline (1,3-D), phthalimide (PM), and phthalic acid, generally in high-boiling non-aqueous solvents at elevated temperatures or electrochemically from phthalonitrile [103,133]. No characterization studies were performed until 1929, when Linstead and co-workers synthesized and elucidated their structure as tetraazabenzoporphyrin [134,135,136,137]. Therefore, Pcs evolved from porphyrins and share some features with their precursors. Moreover, it is a highly efficient PS with a high fluorescence quantum yield and improved spectroscopic properties that are within the therapeutic window for PDT applications in relation to its precursor [138,139]. Overall, most Pcs have been extensively studied as a PS in PDT of cancer due several characteristics, including chemical stability under physiological conditions, optimal selectivity in tumor cells, quick clearance from the body, low cytotoxicity in the dark with high phototoxicity, absorption in the optical transmission window of biological tissues, and high quantum yield of singlet oxygen production [121,140,141].

Among the Pcs, MPcs have been identified as strong inducers of cyto damage and preferentially accumulate in tumor cells, where they stimulate photo damages in various in vitro and in vivo tumor models [142,143]. In vitro, MPcs family members were used to investigate their phototherapeutic activities in various cancer cell lines, including lung, colon, esophageal, cervical, and breast cancer cells [144,145,146,147]. Still, several MPc-based photosensitizers have been used in clinical trials against cancer such as CGP55847 (liposomal formulation of unsubstituted ZnPc), photosens (Pc derivative containing aluminum as the central metal), and photocyanine (isomeric mixture of di-(potassium sulfonate)-di-phthalimidomethyl ZnPc) [148,149]. Finally, among the Pcs evaluated so far in the clinic, Pc4, a silicon-based MPc developed by Vitex/USA, presented the greatest potential against breast, human colon, and ovarian cancers [150,151].

### 3.4. Pcs Optimization of Photophysical Properties and Biocompatibility

**Figure 2:**
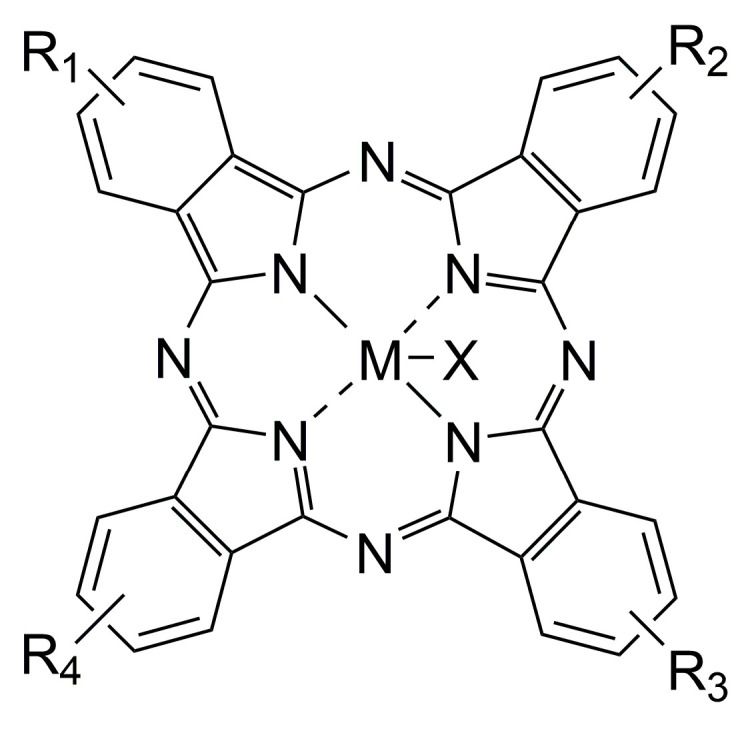
General chemical structure of metallated phthalocyanines (MPcs).

Pcs are large planar aromatic systems composed of four isoindole units linked by nitrogen atoms. Therefore, Pcs present the ability to form stable chelates with at least sixty metal and metalloid ions inserted into the central ring replacing two hydrogens originating the MPcs [36] (Figure 2). Whereas the properties of MPcs vary according to the nature of the central ion, the selection of this ion as well as the synthetic modifications offer numerous options to control their physical properties. Therefore, closed-shell diamagnetic Pcs present higher yields, longer lifetimes of triplet states, and greater tumor retention compared to paramagnetic Pcs, being more suitable for PDT [90,124,152,153,154,155].

As discussed above, unsubstituted Pcs and their metal complexes suffer poor solubility in water and aggregation tendency, limiting their clinical application [32,33,34]. However, due to the chemical structure of PCs, it is possible to introduce various peripheral (macrocycle) and axial (coordination to the central metal ion) substitutions that modulate the tendency for aggregation, pharmacokinetics, biodistribution, and solubility, as well as fine-tuning of NIR absorbance [90,156,157,158,159]. The advantage of increasing solubility through appropriate replacement is that it allows for direct biological administration without the need for an additional vehicle [36].

Typical substitutions to the periphery of the Pcs macrocycle are sulfonation, phosphonation [127,160], glycosylation [161], or carboxylation, or addition of other soluble substituents such as glucose, quaternary amino, hydroxyl, or nitro groups [162]. These peripheral substitutions generate Pcs with increased solubility and reduced tendency to aggregate [90,162]. Suitable ions for axial substitution of the Pcs central ion are Al^3+^ with one or Si^4+^ and Ge^4+^, with two coordination sites. MPcs not substituted with central ions, such as silicon or iron, tend to bond through oxo-bridges. Typical organic substitutes are soluble polymers such as poly(ethylene glycol) or poly(vinyl alcohol). Shorter polymers tend to shorten plasma half-life, but polymers with longer chains significantly prolong plasma half-life compared to unsubstituted MPcs. Axial substitution generally decreases the MPcs aggregation tendency and modulates solubility among other properties [36,163,164]. Additionally, exocyclically metallated Pcs are also an option to improve the properties of Pcs [165].

The solubility and bioavailability of hydrophobic Pcs can be further improved with the use of DDSs. Furthermore, DDSs also have the advantages of improving efficacy and/or reduced toxicity of a PS and delivering them to targeted tissues [36]. Some examples of DDSs used in PCs are liposomes, micelles, nanocapsules, microemulsion, polymeric nanoparticles, solid lipid particles, niosomes, and carbon nanotubes. The use of formulations with DDSs has currently been considered as a very promising strategy to control Pcs’ biological properties [166]. Finally, mitochondria are important regulators of apoptosis. In general, they are responsible from most of the ATP in a cell. Therefore, it is reasonable to design and develop PS that target the mitochondria. PS can be chemically modified (e.g., with triphenylphosphonium derivatives, which insert to the inner membrane of mitochondria) so that they can be actively targeted to mitochondria. Several PS can accumulate in mitochondria owing to their charge in case of positively charged agents. On the other hand, negatively charged agents accumulate in the mitochondria as a result of their hydrophobicity. Furthermore, mitochondrial targeting can also be achieved by synthesizing PS that are attached to mitochondria targeting sequences, which can direct molecules to the mitochondrial matrix [167].

## 4. Pcs as a PS for Cervical Cancer PDT

In this section, we cover the main studies reported in the last five years focusing new Pcs approaches being developed and tested in vitro and/or in tumor-bearing mice and in clinical tests against cervical cancer. More specifically, a review was conducted following a systematic search to identify studies reported between January 2016 and April 2021 focused on “Phthalocyanine” and “Uterine Cervical Neoplasms” in PubMed, Embase, Scopus, and Web of Science databases. To expand the search, references of the original selected articles were evaluated to find papers that could complement this review.

### 4.1. In Vitro Studies Evaluating Pcs on Hela Cells

In the last 5 years, several in vitro studies have been performed using Pcs as a PDT agent against the HeLa cell line. These studies are presented in detail in Table 2. HeLa cells are immortalised cells and the oldest and most commonly used human cell line [168]. The HeLa cell line is derived from cervical cancer cells (adenocarcinoma) taken on 8 February 1951, from Henrietta Lacks, a 31-year-old African-American mother of five, who died of cancer on 4 October 1951 [169]. The cell line is remarkably durable and prolific, which allows it to be used extensively in scientific study [170]. Therefore, in many of the studies that tested different Pcs formulations in HeLa, the main objective was not to evaluate its effectiveness against cervical cancer but against cancer as a whole. However, indirectly, these studies evaluated the activity of these PCs against cervical adenocarcinoma.

#### 4.1.1. MPcs

In general, MPcs were the most studied Pcs against HeLa cells and mostly presented the main objective to increase the solubility and bioavailability of hydrophobic Pcs and their derivatives using various DDSs. It was possible to observe that MPcs formulations demonstrated high photodynamic activity against HeLa cells upon light activation [165,171,175,176,177,178,179,180,181,182,183,185,186,187,188,189,190,192,193,194] while presenting low or absent toxicity in the dark [165,175,176,178,179,181,183,185,188,190,192,193]. Overall, these results show that Pcs formulations were effective in subtoxic doses and had a dose- and time-dependent cytotoxic effect against HeLa cells, highlighting their potential for PDT against cervical cancer.

MPcs formulations successfully entered cells and localized mainly in the cytoplasm of tumor cells [174,177,178,181,182,191,193,194] rather than in cellular membrane [180]. Taken together, these data demonstrate that the Pcs formulations tested exhibited a higher capacity to permeate the cytoplasmic membrane and to internalize in the cytoplasm of HeLa cells. The uptake of the PS by cancer cells is crucial for PDT to be effective since ROS present a short half-life and can only act close to the generation site [104,195]. Hydrophobic molecules can rapidly diffuse into plasmatic membranes, while more polar drugs tend to be internalized via endocytosis or assisted transport by serum lipids and proteins. However, the exact mechanisms of MPcs cellular uptake against HeLa cells are still unclear and require further investigation.

The PDT efficiency depends on the illumination conditions, the chemical properties, and the intra-tumoral localization of the PS. Still, the type of photodamage in cells loaded with a PS and illuminated depends on the precise subcellular location of the PS. Thus, understanding the location of the PS is an important principle to consider the most effective PS for each application [104,195]. The subcellular localization of MPcs in Hela cells included mitochondria [174,193] and lysosomes [171,172,174,175,177,178,194] but rarely at the nucleus [165]. This evidence highlights the potential of MPcs as a PS for PDT against cervical adenocarcinoma, since most potent PS are usually localized in mitochondria and/or lysosomes [196].

It is well known that the production of ROS at mitochondrial, lysosomal, or ER loci can directly initiate apoptotic cell death [111]. Accordingly, the HeLa cells death pathway induced by MPcs was apoptosis [171,177,180,185,187,192], compatible with its sub-localization in the mitochondria and lysosomes. These results are very promising as overall apoptosis is considered the most desired mechanism of programmed cell death in PDT due to the absence of side effects compared to necrosis. High doses of PDT and/or uncontrolled photodamage lead to the uncontrolled release of biomolecules of unscheduled cell death into the extracellular space, initiating an inflammatory response in the surrounding tissue. For this reason, necrosis is generally seen as an undesirable mechanism. On the other hand, specific photodamage at adequate doses of PDT can be lethal to cells without harming surrounding healthy cells [196].

MPcs also exerted long-term dose-dependent phototoxic effects against HeLa cells according to the study of Balçik-Erçi et al. 2020 [177]. This effect was obtained with a peripherally biotin substituted zinc(II) Pc that reduced the cell colony capacity, which reflects the decrease in long-term cell proliferation. In general, the results of in vitro studies were promising and suggested that different formulations of MPcs are among the best currently-used PS of cervical adenocarcinoma PDT and their use in clinical studies should be encouraged for prospective means of managing this cancer.

Additionally, the combination of PDT and chemotherapy can induce synergistic therapeutic effects: the PS can overcome multidrug resistance, while the chemotherapeutic drugs can address the limitations of light penetration and hypoxia-related resistance in PDT and enhance the sensitivity of cancer cells to ROS [197]. Recently, Ma et al. 2018 [182] developed a ZnPC-soybean phospholipid complex based on DDSs with doxorubicin (Dox). In vitro evaluations indicated that MTX-decorated self-assembled ZnPc-SPC complex nanoparticles (NPs), referred as DZSM, presented high selectivity for FRα over-expressed tumor cells as HeLa cells; significant multiphase PDT effect; significant cytotoxicity; and great synergetic therapy potential. Additionally, Ma et al. 2018 [183] developed novel DOX/ZnPc co-loaded mesoporous silica (MSNs)@ calcium phosphate (CaP)@PEGylated liposome NPs. In vitro assays with HeLa cells indicated that CaP could not only achieve the controllable release of PS triggered by pH but also promote its cellular uptake and induce apoptosis. According to the authors, the MSNs@CaP@PEGylated liposomes could serve as a promising nanoplatform for cancer treatment by synergic chemo-PDT and superior tumor-targeting ability.

The results of both studies highlight that MPcs could serve as a promising multifunctional platform in anticancer treatment by synergic chemo-PDT and superior tumor-targeting ability.

#### 4.1.2. Silicon Pcs (SiPcs)

Two recent studies investigated the in vitro activity of silicon Pcs (SiPcs) formulations against HeLa cells. Unlike most other Pcs, SiPcs possess two additional axial bonds that reduce aggregation in solution and can be synthetically tailored, thereby creating further scope for modulation of optical, chemical, and electronic properties [198]. The results of both studies [173,184] were very promising as they showed that SiPcs presented high photo cytotoxicity against HeLa cells and were non-cytotoxic/low-cytotoxic in the dark.

Finally, as far as we know, little has been studied about photobleaching in metal-free Pc and MPC formulations against HeLa cells or other cervical cancer cell lineages.

Although the results presented in Table 2 are promising, some important points should be highlighted:

1. Considering that the different studies included in Table 2 used different concentrations of MPCs, different light exposure times, and different light sources, it is very difficult to compare the results obtained between these studies.
2. Still, for the same reasons, it is very difficult to assess which formulation presented the best therapeutic efficacy.
3. The number of recent studies evaluating the combination of PDT and chemotherapy is very small and restricted to doxorubicin, limiting interpretations of its real benefit.

### 4.2. Both In Vitro and In Tumor-Bearing Mice Pcs Studies Based on HeLa Cells

A small number of studies evaluated Pcs effects against HeLa cells in vitro and in vivo. Regarding in vivo data, these were performed in tumor-bearing mice with HeLa cells [29,199,200,201] (Table 3). Briefly, Wang et al. 2019 [201] proposed that the therapeutic activity of the MPc (sulfonated Al-phthalocyanine-AlPcS) can be efficiently excited via plasmonic-resonance energy transfer from the two-photon excited gold nanobipyramids (GBPs) and further generates cytotoxic singlet oxygen for cancer eradication. In vitro, after incubation with GBP-AlPcS and exposure to laser irradiation, approximately 80% of HeLa-cell mortality rate was achieved at GBP-AlPcS. In comparison, the compound presented low dark cytotoxicity. In the study conducted by Li et al. 2019 [29], other MPc (zinc II Pc) derivative entrapped mesoporous silica NPs (MSNs) and a wrapping DNA (O1) (PcC4-MSN-514 O1) displayed in vitro selective phototoxicity against HeLa cells. In vivo, with systemic administration of PcC4-MSN-O1, there was accumulation in HeLa tumors of xenograft-bearing mice. After laser irradiation, tumor growth was inhibited and apoptosis was induced. Moreover, the time-modulated activation process in tumors and the relatively fast excretion of PcC4-MSN-O1 indicated its advantages in reducing potential side effects. Li et al. 2019 [199] introduced two polyethylene glycol (PEG) linkers on SiPc to synthesize a new water-soluble and tumor-targeting photosensitizer compound. In vitro, this compound accumulated in biotin receptor (BR)-positive Hela cells through BR-mediated internalization. In vivo, SiPc preferentially accumulated in the tumor tissue of the tumor-bearing mice. Furthermore, SiPc significantly depressed tumor progression after irradiation. Finally, Liang et al. 2021 [200] tested FA-TiO2-Pc in conjunction with folic acid (FA), a tumor-targeting agent. In vitro, FA-TiO2-Pc presented high therapeutic efficiency at low concentration and with a short incubation time, under one-photon excitation. In mice with HeLa xenograft tumors, FA-TiO2-Pc led to decreased tumor growth with minimal side effects, even at low concentrations and low light irradiation.

Therefore, the results of these studies highlight that the tested MPcs formulations are very promising for cervical cancer PDT, including in vivo and presented great potential for future use in clinical studies. However, the limitations highlighted above for the studies included in Table 2 can be applied to the studies shown in Table 3. Therefore, additional interpretations of these studies are greatly impaired.

### 4.3. Studies Evaluating Pcs in Other Cervical Cancer Cells than Hela

In the last 5 years, only one study evaluated Pcs in other cervical cancer cell lines in addition to HeLa [17]. This study evaluated the effectiveness of PDT using Silicon phthalocyanine 4 (Pc4) in vitro and in vivo against human cervical cancer cells CaSki (squamous cell carcinoma drive cell line, HPV-16 about 600 copies per cell, and HPV-18) and ME-180 (squamous cell carcinoma drive cell line, HPV DNA with greater homology to HPV-68 than HPV-18). Specifically, cell growth and cytotoxicity were measured in vitro using a methyl thiazole tetrazolium assay. Pc4 cellular uptake and intracellular distribution were determined. For in vitro Pc4 PDT, cells were irradiated at 670 nm at a fluence of 2.5 J/cm^2^. SCID mice (female C.B-17 mice) were implanted with CaSki and ME-180 cells both subcutaneously and intracervical. Forty-eight hours after Pc4 administration, the tumors were irradiated at 75 and 150 J/cm^2^, for 4 min. In vitro, Pc4 itself was relatively non-toxic and Pc4 PDT was effective in killing cervical cancer cells grown as either spheroids or monolayers. Additionally, in both cervical cancer cell lines, the subcellular distribution of Pc4 occurred in the cytoplasm, lysosomes, RE, and mitochondria. In vivo, intracervical tumors became necrotic after Pc4 PDT. In general, this study highlighted the in vitro and in vivo potential of PDT with Pcs against invasive cervical cancer due to HPV-16, the most prevalent HPV type in cervical cancer. Furthermore, these results occurred more specifically in squamous cell carcinoma, which is the most common type of cervical cancer worldwide (approximately 70% of total) [40].

### 4.4. Clinical Studies

As far as we know, only a recent study performed the clinical treatment of CIN with Pcs [24]. PDT was performed using a chitosan NP containing chlorocyan-aluminum Pc in patients with CIN1 and CIN2. The compound was applied on the surface of the cervix of each selected patient with CIN1 (*n* = 11) or CIN2 (*n* = 1), which was then illuminated after 30 min with a red laser lighting (minimum dose of 200 J, power of 1.20 W, irradiance of 0.30 W·cm^−2^, and fluency of 50 J·cm^−2^). Among the 12 patients treated primarily, 11 (91.7%) presented negative cervical cytology in the first evaluation after treatment, but 1 (8.3%) presented no therapeutic benefit, even after reapplication. Two of the patients who presented a good initial therapeutic response had relapse, but evolved to cytological remission after a new round of PDT, remaining negative until the last follow-up. No important side effects were observed in any of the patients. According to the authors, the trial demonstrated that the treatment of CIN1 and CIN2 lesions using PDT with CNP-AlClPc is feasible and safe. However, they suggested large randomized clinical trials to establish efficacy.

To date, Pcs have not been used in clinical studies for the PDT of invasive cervical cancer. According to clinicaltrials.gov, there is only one published study of a clinical trial involving cervical cancer and PDT [202].

## 5. Conclusions

Throughout this review, we highlighted that the main topics in Pcs as agents for PDT against cervical cancer are solubility, targeting capacity, and therapeutic efficiency. At a chemical level, it is concluded that Pcs amphiphilic character leads to higher efficiency in vitro and in vivo. Furthermore, the phototoxic potential as well as its cellular uptake and internalization seem to be improved, making it difficult to stack it via axial ligation and inclusion of positive charge(s). Additionally, the latest in vitro and in vivo studies of Pcs against cervical cancer reported in this review strongly suggest that the application of nanotechnologies as DDSs allowed for increased PDT efficacy and reduced side-effects associated with the Pcs administration, which shows promise for cervical cancer treatment.

Hence, only considering their chemistry, Pcs exhibit this flexibility, enabling further screening and investigations, which raises hope for cervical cancer treatment. However, we highlight some important considerations as follows:

- Current evidence indicates that despite the increasing number of studies with a growing number of different Pcs formulations and their generally increased number of favorable aspects as mainly related to in vitro low effective concentration (mainly against the tumour cell line), low dark toxicity, increased photo cytotoxicity, and cellular uptake in a dose-dependent manner for cervical cancer, only a few Pcs were evaluated in cervical cancer cell lines other than HeLa. As a result, most studies assessed the activity of different Pcs formulations against cervical adenocarcinoma and not against squamous cervical cancer, which is the most common type of cervical cancer worldwide (approximately 70% of total) [40]. Additionally, few preclinical animal studies and no clinical studies with Pcs in invasive cervical cancer have been performed to date.
- Regarding pre-clinical animal models [203], there are only a few studies in tumor-bearing mice xenograft tumors based on HeLa cells. Therefore, there is an urgent need for more in vivo studies in tumor-bearing mice and primates based on HeLa cells and in other cervical cancer lineages to continue studies on the effectiveness of different Pc formulations against cervical cancer.
- Indeed, our review showed that in the field of Pcs in cervical cancer, the photophysical and photochemical properties, subcellular localization, phototoxic activity, mechanisms of cell death, and improved targeting to tumor tissues have been the main topics explored. However, up to this moment, there is an immediate need to increase the number of clinical trials evaluating Pcs in CIN and invasive cervical cancer. As a result, it would be possible to confirm preclinical studies and orient future research.
- Additionally, we showed that the combination of PDT with MPcs and chemotherapy using Dox can induce synergistic therapeutic effects, highlighting that MPcs could serve as a promising multifunctional platform in anticancer treatment by synergic chemo-PDT and superior tumor-targeting ability. However, the number of studies with this approach is still limited, so we strongly suggest implementing research in this area.
- Finally, the search for new possible mechanisms of action in different cervical cancer cells may consequently contribute to novel applications for single or combined Pcs treatments to CIN and invasive cervical cancer.

In conclusion, Pcs are promising as pharmaceutical entities for anti-cervical cancer agents. Major research requirements are needed to encourage studies evaluating Pcs in cervical cancer cell lines other than HeLa and in squamous cervical cancer type; increase preclinical animal studies and clinical trials evaluating Pcs in CIN and invasive cervical cancer to confirm preclinical in vitro studies and orientate future research; implement research in by combining PDT with Pcs and chemotherapy; and understand the new exact mode of actions of Pcs in different cervical cancer cells. Despite all the drawbacks and limitations mentioned above, the authors firmly believe that Pc-based formulations could emerge as a privileged scaffold for the establishment of lead compounds for PDT against cervical cancer. A deeper understanding of the effects of PDT with Pcs could enable the future improvement of currently used protocols and the treatment of different cervical cancer types.

## Figures and Tables

**Table 1:** Main human immortalized cervical cancer cell lines available for in vitro studies from uterine cervix tissues.

Cell Line	Histologic Type	Donor Age	HPV	p53	pRb
**HeLa (ATCC^®^ CCL-2™)**	Adenocarcinoma	31	HPV-18	Positive (low)	Positive (normal)
**SiHa (ATCC^®^ HTB-35™)**	Squamous cell carcinoma	55	HPV-16, 1 to 2 copies per cell	Positive	Positive
**CasKi (ATCC^®^ CRM-CRL-1550™)**	Squamous cell carcinoma	40	HPV-16, about 600 copies per cell, and HPV-18	NA	NA
**C-33A (ATCC^®^ HTB-31™)**	Squamous cell carcinoma	66	Negative	Positive	Positive
**MS751 (ATCC^®^ HTB-34™)**	Squamous cell carcinoma	47	HPV-18 HPV-45	NA	NA
**ME-180 (ATCC^®^ HTB-33™)**	Squamous cell carcinoma	66	HPV DNA with higher homology to HPV-68 than HPV-18	Positive	Positive
**C-4 I (ATCC^®^ CRL-1594™)**	Squamous cell carcinoma	41	HPV-18	NA	NA

NA: not applicable; pRb, retinoblastoma protein; p53, protein 53; and HPV, human papillomavirus.

**Table 2:** Main recent (2016–2021) in vitro studies evaluating different formulations of phthalocyanines in HeLa cells.

Title	Reference	PS/Concentration/ Time of Exposure to PS	Light Source/ Wavelength/Fluence/ Power-Density/ Time of Exposure	Main Outcomes
Exocyclically metallated tetrapyridinoporphyrazine as a potential photosensitizer for photodynamic therapy	[165]	Exocyclically metallated tetrapyridinoporphyrazine [tetrakis-(trans-Pt(NH3)2Cl)-tetra(3,4-pyrido)porphyrazine-zinc(II)](NO3)4, 1.6 μM, and 4 h	600 nm, 5.8 mW/cm^−2^, 6.96 J/cm^−2^, and 20 min	Single digit micromolar concentrations are able to induce photocytotoxicity while maintaining low toxicity in the dark. The compound mainly accumulates in the nucleus, suggesting that interacts with DNA, leading to subsequent DNA damage and resulting in photocytotoxicity.
Peripherally crowded cationic phthalocyanines as efficient photosensitizers for photodynamic therapy	[171]	Zinc phthalocyanine bearing four or eight bulky 2,6-di(pyridin-3-yl)phenoxy substituents, 0–10 μM, and 12 h	570 nm, 12.4 mW/cm^2^, 11.2 J/cm^2^, and 15 min	High photodynamic activity against cancer cells while maintaining low toxicity in the dark. Localization in the lysosomes, inducing an apoptotic cell death pathway with secondary necrosis.
Facile synthesis of cyclic peptide−phthalocyanine conjugates for epidermal growth factor receptor-targeted photodynamic therapy	[172]	Cyclic peptide-conjugated zinc(II) phthalocyanine, 0–60 nM, and 2 h	610 nm, 23 mW/cm^2^, 28 J/cm^2^, and 20 min	The intensity of cell uptake in EGFR-positive HT29 and HCT116 cells is up to 25 times higher than against EGFR-negative HeLa and HEK293 cells. This conjugate also shows high photo cytotoxicity for HT29 and HCT116 cells.
Synthesis and photodynamic activities of novel silicon(IV) phthalocyanines axially substituted	[173]	Quaternized cationic silicon(IV) phthalocyanine (SiPc) derivatives, 0–10 μM, and 24 h	680 ± 10 nm, 2/J cm^2^, and NR	High photodynamic activity against cancer cells while maintaining low toxicity in the dark.
Effects of zinc porphyrin and zinc phthalocyanine derivatives in photodynamic anticancer therapy under different partial pressures of oxygen in vitro	[174]	Disulphonated zinc phthalocyanine (ZnPcS_2_) and tetrasulphonated zinc tetraphenylporphyrin (ZnTPPS_4_), 0–10 μM, and 24 h	ZnTPPS_4_: 415 ± 10 nm ZnPcS_2_: 660 ± 15 nm; 7 mW/cm^2^, 4.2 J/cm^2^, and 10 min	ZnTPPS_4_ was internalized in the cytosol and lysosomes, whereas ZnPcS_2_ was attached to membrane structures and was photodynamically effective at a minimal level of oxygen, with a higher effect on mitochondrial respiration.
Assessing amphiphilic ABAB Zn(II) phthalocyanines with enhanced photosensitization abilities in in vitro photodynamic therapy studies against cancer	[175]	Triethylene glycol (TEG)-containing Zn(II)Pcs, namely, ABAB-1, A3B-1, and A4-1	ABAB-1, A3B-1, and A4-1; 637 nm ± 17 nm; different red light doses (3, 6 and 9 J/cm^2^), NR	ABAB-1 and A3B-1 presented high photodynamic activity on cancer cells while maintaining low toxicity in the dark.
In vitro bioeffects of polyelectrolyte multilayer microcapsules post-loaded with water-soluble cationic photosensitizer	[176]	Dextran sulfate (DS) and poly-l-arginine (PArg) PMC ([DS/PAgr]4) capsules loaded with zinc phthalocyanine choline derivative (cholosens), NR	-	High drug release rate, internalization, light toxicities, and low dark effects.
Improved targeting for photodynamic therapy via a biotin–phthalocyanine conjugate: synthesis, photophysical and photochemical measurements, and in vitro cytotoxicity assay	[177]	Peripherally biotin-substituted zinc(II) phthalocyanine (Pc_2_), 0.25–5 μM, and 24 h	690 ± 10 nm, 1/J cm^2^ and 2/J cm^2^, and NR	The biotin-conjugated zinc(II) phthalocyanine derivative presented a higher cytotoxic effect than the amino functionalized zinc(II) phthalocyanine derivative. Pcs were located in the cytoplasm, leading to cell death by apoptosis and reduction of colony capacity after PDT.
Cationic versus anionic phthalocyanines for photodynamic therapy: what a difference the charge makes	[178]	Anionic and cationic zinc(II) phthalocyanines, 1–10 mM, and 12 h	570 nm, 12.4 mW/cm^2^, 11.2 J/cm^2^, and 15 min	Hydrophilic compounds were localized into lysosomes and amphiphilic compounds were also detected in the cellular membrane. Hydrophilic cationic Pcs were relocalized into the cytoplasm upon irradiation and damaged the nuclear membrane. A high dose of Pcs induced morphological changes and phototoxicity.
Multiple functions integrated inside a single molecule for amplification of photodynamic therapy activity	[179]	Arg and Lys zinc phthalocyanines, 0–10 μM, and 48 h	665 nm, 5 W, 0.4 W/cm^2^, and 4 min	The phototoxic effects were more accentuated and the percentage of apoptotic cells was higher in the cells treated with Arg-ZnPc.
Apomyoglobin is an efficient carrier for zinc phthalocyanine in photodynamic therapy of tumors	[180]	Zinc phthalocyanine carried by apomyoglobin, 500 nM, and NR	647 nm, 2.5 mW, 130 mW/cm^2^, 40 J/cm^2^, and 5 min	The uptake of ZnPc by cells was efficient, with no dark toxicity. When illuminated, a moderate fluence and low concentrations were sufficient to induce extensive cell death.
Novel theranostic zinc phthalocyanine–phospholipid complex self-assembled nanoparticles for imaging-guided targeted photodynamic treatment with controllable ROS production and shape-assisted enhanced cellular uptake	[181]	Zinc phthalocyanine-soybean phosphatidylcholine (ZnPc-SPC) complex, 0.3–10 µg/mL, and 12 or 24 h	630 nm, NR, and 5 min	Pcs could target folate receptors-overexpressed cancer cells and internalized in the cytoplasm. Apoptosis rate increased after PDT.
Zinc phthalocyanine-soybean phospholipid complex based drug carrier for switchable photoacoustic/fluorescence image, multiphase photothermal/ photodynamic treatment and synergetic therapy	[182]	Zinc phthalocyanine-soybean phospholipid complex with doxorubicin (DZSM), 0.3–10 µg/mL, and 12 h	638 nm, 1 W/cm^2^, NR, and 5 min	ZnPc was distributed in the cytoplasm. Dox was almost located in the nucleus. DZSM presented high selectivity for FRα over-expressed tumor cells as HeLa, excellent switchable image, significant multiphase photothermal therapy (PTT)/PDT effect, and great synergetic therapy potential, leading to notable inhibition of tumor growth.
Novel core-interlayer-shell DOX/ZnPc Co-loaded MSNs@ pH-sensitive CaP@PEGylated liposome for enhanced synergetic chemo-photodynamic therapy	[183]	DOX/ZnPc co-loaded MSNs@CaP@PEGylated liposome, NR, and 24 h	630 nm, 0.05 W/cm^2^, NR, and 5 min	ZnPc in the nanoparticles successfully produced the intracellular singlet oxygen under the light that could eventually induce the cytotoxicity of PDT in the cells and could be a promising candidate for PDT besides serving as a chemotherapeutic agent.
Silicon(IV) phthalocyanine-biotin conjugates: synthesis, photo physicochemical properties and in vitro biological activity for photodynamic therapy	[184]	Axially biotin substituted silicon(IV) phthalocyanine, 0–10 μM, and 24 h	NR, 1 J/cm^2^ or 2 J/cm^2^, and NR	Both axially mono- and bis-biotin substituted silicon(IV) phthalocyanines presented high photo cytotoxicity against HeLa cancer cells with the cell survival degree ranging from 13% to 50%. The photosensitivity and the intensity of damage were found to be directly related to the concentration of the used photosensitizers.
Positively charged phthalocyanine-arginine conjugates as efficient photosensitizer for photodynamic therapy	[185]	Arginine substituted zinc phthalocyanines (ArgEZnPc and ArgZnPc), 0–4 µM, and 4 h	665 nm, 96 mW/cm^2^, 28.8 J/cm^2^, 5 min, and NR	ArgEZnPc presented higher cellular uptake, high water solubility and ROSs generation ability. HeLa cells showed shrinkage and cell scatter, membrane deformation, and chromatin damage. ArgEZnPc can target the lysosomes and exhibited high cytotoxicity.
Intracellular uptake and fluorescence imaging potential in tumor cell of zinc phthalocyanine	[186]	Zinc phthalocyanine (ZnPc), 10–90 mM, and 24 h	NR	The IC_50_ values were observed to be 35 mM in HeLa cells, maximum uptake was determined at 6 h, and the uptake was decreased at 24 h.
In vitro effects of photodynamic therapy induced by chloro aluminum phthalocyanine nanoemulsion	[187]	Chloroaluminum phthalocyanine nanoemulsion (ClAlPc/NE) or MX+ ClAlPc/NE (methoxyamine), NR, and 3 h	670 nm, 0.1, 0.5 and 1.0 J/cm^2^, and NR	A dose-dependent cell death reduced clonogenic survival rates, and sub-G1 accumulation and apoptosis induction were observed in HeLa cells. MX increased PDT effects.
Synthesis of asymmetric zinc(II) phthalocyanines with two different functional groups and spectroscopic properties and photodynamic activity for photodynamic therapy	[188]	Zinc(II) phthalocyanine functionalized, 0–10 µM, and 24 h	690 ± 10 nm, 2 J/cm^2^, and NR	The photodynamic efficiency is micromolar. Biotin conjugated zinc(II) phthalocyanine displayed a higher photo cytotoxicity relative to amino phthalocyanine, probably attributed to its high triplet quantum yield of ^1^O_2_.
Triblock copolymers encapsulated poly (aryl benzyl ether) dendrimer zinc(II) phthalocyanine nanoparticles for enhancement in vitro photodynamic efficacy	[189]	Zinc (II) phthalocyanines nanoparticles with triblock copolymer (G2-DPcZn), 0.02–10 µM, and 24 h	670 nm, 25, 50, and 100 mW/cm^2^, 0–6 J/cm^2^, and 2 min	The nanocarriers enhanced intracellular uptake, phototoxicity, and ROS production. The nanoparticle surface with positive charge seems to localize G2-DPcZn in mitochondria.
Cyclodextrin type dependent host-guest interaction mode with phthalocyanine and their influence on photodynamic activity against cancer	[190]	Phthalocyanines (Pc) with cyclodextrins (CDs), 5 µM, and 4 h	665 nm, NR, and 5 min.	The aggregation degree of Pcs was decreased, the water solubility and photodynamic activity were increased. The cellular uptake and ROS generation efficiency of (-CD)4-ZnPc was higher. PDT induced morphology changes, such as chromatin condensation, shrinkage, and fragmentation.
Drug delivery function of carboxymethyl-β-cyclodextrin modified upconversion nanoparticles for adamantine phthalocyanine and their NIR-triggered cancer treatment	[191]	UCNP/COOH-β-CD/Ad-ZnPc, 2–8 μM, and 4 h	980 nm, NR, and 1–4 sessions of 2 min	The complex was mainly located in the cytoplasm and after irradiation induced morphology changes, such as chromatin condensation, shrinkage, and fragmentation. The UCNPs and Ad-ZnPc and UCNP/COOH-β-CD/Ad-ZnPc treated cell survival percent sharply decreased with the increasing of drug concentration and light dose.
Phototoxicity of liposomal Zn- and Al-phthalocyanine against cervical and oral squamous cell carcinoma cells in vitro	[192]	Liposomal Zn- and Al-phthalocyanine, 0.1–1 μM, and 24 h	ZnPc: 350–800 nm, 43.2 J/cm^2^; AlPc: 690 nm, 3.6 J/cm^2^; 20 min	Liposome-embedded ZnPc and AlPc were more effective than free ZnPc and AlPc in reducing cell viability. HeLa cervical adenocarcinoma cells were more sensitive to AlPc.
Photodynamic therapy and nuclear imaging activities of zinc phthalocyanine-integrated TiO_2_ nanoparticles in breast and cervical tumors	[193]	Zinc phthalocyanine integrated to the TiO_2_ nanoparticle (ZnPc- TiO_2_), 1–6.25 μM, and 3h	LED light source, 10 mW/cm^2^, 30, 60, and 90 J/cm^2^, and NR	TiO_2_ nanoparticles increased the cytotoxicity of ZnPc in the HeLa cell line. Phototoxic effects increased depending on the dose of light. Cellular localization of the Pcs was found especially in cytoplasm but not nuclei.
Cervical cancer cells (HeLa) response to photodynamic therapy using a zinc phthalocyanine photosensitizer	[194]	Sulphonated zinc phthalocyanine PS (ZnPcSmix), 0.25–1 μM, and 24 h	673 nm diode laser, 96 mW, 2, 4 and 8 J/cm^2^, and NR	The PS was located in the cytoplasm and perinuclear region of HeLa cells. PDT induced dose-dependent structural changes, with decreased cell viability and proliferation, as well as membrane damage.

PS, photosensitizer; NR, not reported.

**Table 3:** Recent (2016–2021) in vitro and in vivo studies evaluating different formulations of phthalocyanines on HeLa cells.

Title	Reference	Animal Model	PS/Concentration/ Time of Exposure to PS	Light Source/Wavelength/Fluence/Power/Density/Time of Exposure	Main In Vitro Outcomes	Main In Vivo Outcomes
Functional titanium dioxide nanoparticle conjugated with phthalocyanine and folic acid as a promising photosensitizer for targeted photodynamic therapy in vitro and in vivo	[200]	Female BALB/c nude mice inoculated subcutaneously on the right armpit with 100 μL HeLa cells (1 × 10^7^ cells) in PBS.	TiO_2_ nanoparticle conjugated with folic acid (FA), and Al (III) phthalocyanine chloride tetrasulfonic acid (FA-TiO_2_-Pc), 0.52 μmol/kg, 6 h	420–800 nm, 0.75 W/cm^2^, 10 min.	FA-TiO_2_-Pc presented high therapeutic drug efficiency at a low concentration dose and short incubation time under one-photon excitation.	Tumor growth of the FA-TiO_2_-Pc treated mice was significantly inhibited. The survival rates were 100%, and no significant physiological morphology changes were observed in heart, liver, spleen, lungs, and kidney, indicating no toxic effects after treatment.
A biotin receptor-targeted silicon(IV) phthalocyanine for in vivo tumor imaging and photodynamic therapy	[199]	Female BALB/c nude mice inoculated subcutaneously on the right foreleg armpit with 100 μL of PBS containing Hela cells (1 × 10^7^)	Biotin receptor-targeted silicon(IV) phthalocyanine, 2 μmol/kg, 2 h	670 nm, 10 mW/cm^2^, 30 min	High photodynamic activity on cancer cells while maintaining low toxicity in the dark.	The compound specifically accumulated in tumor tissue through the biotin receptor-mediated process, allowing for the targeted imaging of the tumor tissue in vivo. Moreover, under-irradiation induced clear necrosis of the tumor tissues and the tumor’s growth was inhibited.
Sequential protein-responsive nanophotosensitizer complex for enhancing tumor-specific therapy	[29]	Male NOD-SCID mice injected subcutaneously with HeLa cells (2 × 10^7^ cells)	zinc(II) phthalocyanine derivative entrapped mesoporous silica nanoparticles (MSNs) and a wrapping DNA (O1) (PcC4-MSN-O1), 200 μM, 24 h	670 nm, 0.5 W/cm^2^, 20 min	PcC4-MSN-O1 displayed selective phototoxicity against HeLa over normal cells (HEK-293).	There was an accumulation in HeLa tumors of xenograft-bearing mice, and irradiation induced the inhibition of tumor growth and apoptosis. The time-modulated activation process in tumors and the relatively fast excretion of PcC4-MSN-O1 indicated its advantages in reducing potential side effects.
AlPcS-loaded gold nanobipyramids with high two-photon efficiency for photodynamic therapy in vivo	[201]	Nude mice inoculated subcutaneously with HeLa cells	Sulfonated Al-phthalocyanine (AlPcS)-loaded by gold nanobipyramids, 5 nM, 2 h	800 nm 2.8 W/cm^2^., 30 min	High photodynamic activity against cancer cells.	An evident inhibition in tumor growth and extensive necrosis was observed in mice after PDT treatment with GBP-AlPcS. Moreover, no side effect or toxicity to normal tissues was observed.

PS, photosensitizer.
